# Establishment probability in newly founded populations

**DOI:** 10.1186/1756-0500-5-313

**Published:** 2012-06-20

**Authors:** Markus Gusset, Michael S Müller, Volker Grimm

**Affiliations:** 1 Wildlife Conservation Research Unit, Department of Zoology, University of Oxford, Abingdon, OX13 5QL, UK; 2 Department of Ecological Modelling, UFZ, Helmholtz Centre for Environmental Research – UFZ, 04318, Leipzig, Germany

**Keywords:** Allee effect, Establishment probability, Extinction risk, *Lycaon pictus*, Population viability, Reintroduction effort, Wissel plot

## Abstract

**Background:**

Establishment success in newly founded populations relies on reaching the established phase, which is defined by characteristic fluctuations of the population’s state variables. Stochastic population models can be used to quantify the establishment probability of newly founded populations; however, so far no simple but robust method for doing so existed. To determine a critical initial number of individuals that need to be released to reach the established phase, we used a novel application of the “Wissel plot”, where –ln(1 – *P*_0_(*t*)) is plotted against time *t*. This plot is based on the equation $P_{0}\left(t\right)=\;1–c_{1}e^{–\omega_{1t}}$, which relates the probability of extinction by time *t*, *P*_0_(*t*), to two constants: *c*_1_ describes the probability of a newly founded population to reach the established phase, whereas *ω*_1_ describes the population’s probability of extinction per short time interval once established.

**Results:**

For illustration, we applied the method to a previously developed stochastic population model of the endangered African wild dog (*Lycaon pictus*). A newly founded population reaches the established phase if the intercept of the (extrapolated) linear parts of the “Wissel plot” with the *y*-axis, which is –ln(*c*_1_), is negative. For wild dogs in our model, this is the case if a critical initial number of four packs, consisting of eight individuals each, are released.

**Conclusions:**

The method we present to quantify the establishment probability of newly founded populations is generic and inferences thus are transferable to other systems across the field of conservation biology. In contrast to other methods, our approach disaggregates the components of a population’s viability by distinguishing establishment from persistence.

## Background

Trying to (re)establish populations by releasing individuals into suitable habitat is an important element of modern conservation practice. The success of such release attempts depends largely on two factors, namely the newly founded population reaching the established phase and, once this stage is reached, maintaining itself in the release area (on the importance of this distinction, see [1]). The established phase is defined by characteristic fluctuations of the population’s state variables (e.g. number of individuals or age structure), in which case population dynamics is no longer affected by initial conditions [1]. Establishment success often depends on how many individuals or groups are released, but high post-release dispersal can create a disparity between release population size and the effective initial size of a newly founded population [2]. Establishment does not imply persistence, as the ecological capacity of the release area may be small and environmental stochasticity large (for a visualization of the established phase, see [3]).

In a previous study [4], we used a stochastic population model of the endangered African wild dog (*Lycaon pictus*) to quantify the critical initial number of packs (two) and individuals per pack (six) necessary for a reintroduced population of this species to maintain itself in the release area. Persistence was virtually impossible, unless the population was frequently supplemented. Given sufficient prey, the intervals between artificially adding a pack seem to be the most important factor governing the persistence of a small, reintroduced wild dog population. However, [4] focused on persistence and post-release management, but not on the probability that the newly founded population reaches the established phase in the first place.

Two important questions arising in any release attempt [2] thus are: (1) How many individuals or groups should be released so that population establishment is more or less guaranteed? (2) In the case of no supplementation, how is persistence of an established population affected by post-release dispersal (i.e. loss of dispersers due to emigration from the release area)? Here, we present a method to quantify the establishment probability of newly founded populations, using attempts to reintroduce wild dogs for illustration. In contrast to other methods, our approach disaggregates the components of a population’s viability by distinguishing establishment from persistence.

### Modelling approach

To tackle these questions, we used our previously developed individual-based model for wild dogs [4]. Individual-based models enable us to explore how population characteristics emerge from the ways in which individuals behave and interact with each other [5]. The model includes social structure and behaviour, but nevertheless is conceptually simple. It is parameterized with data from a 25-year field study in Hluhluwe-iMfolozi Park, South Africa [6].

In short, the model was designed to predict the probability of small, reintroduced populations of wild dogs establishing themselves and persisting in the release area under various levels of reintroduction effort (for details, see [4]). In contrast to [4], no post-release management interventions occurred in the present application of the model and disperser groups were simulated to leave the release area with various probabilities.

Our validation procedure ensured that the model correctly captures internal relationships between variables and to some degree the internal organization of the real system (see [4]). This suggests that the model is appropriate for its intended purpose, as it could reproduce multiple output patterns observed at different hierarchical levels of the system [7], which were not imposed onto the model but emerged from interactions between the simulated individuals, packs and disperser groups.

The robustness of the model was evaluated by conventional local sensitivity analysis of all model parameters, where each parameter was varied separately by ±10% of its mean value (rounded to integer if required). The analysis showed a moderate sensitivity *s* of the individual parameters (*s* = ratio of the relative change in the intrinsic mean time to extinction *T*_m_ to the relative change in parameter value) (Table 1).

**Table 1:** Model parameters, reference values and results of the local sensitivity analysis for a reintroduced wild dog population (initial condition: number of packs = 4; probability for a disperser group to leave release area = 0.4)

Parameter	Reference value	Sensitivity	
		+10% of parameter value	–10%of parameter value
Reproduction in newly formed packs (*p*)	0.33	2.07	–0.22
Reproduction in established packs (*p*)	0.66	7.62	–4.24
Pack size (*v*)	8.1 ± 1.1	0.20	–0.37
Litter size (*v*)	7.9 ± 0.8	3.63	–3.16
Primary sex ratio (*p*)	0.55 ± 0.06	–0.62	2.11
Ecological capacity (*v*)	62	1.76	–0.16
Density dependence threshold (*v*)	31*	3.23	–2.82
Dispersal in males (*p*)	0.80*	–0.96	1.52
Dispersal in females (*p*)	0.90*	–1.08	2.48
Disperser group size threshold (*v*)	2*	0.27	–0.14
Pack formation (*p*)	0.64	0.50	–0.12
Dominant displacement (*p*)	0.20	0.46	–0.12
Mortality in male pups (*p*)	0.07 ± 0.06	–0.53	0.96
Mortality in female pups (*p*)	0.16 ± 0.14	–0.37	0.34
Mortality in yearling males (*p*)	0.29 ± 0.14	–0.53	1.67
Mortality in yearling females (*p*)	0.20 ± 0.20	–0.46	0.00
Mortality in young adult males (*p*)	0.17 ± 0.08	–1.39	1.83
Mortality in young adult females (*p*)	0.01 ± 0.01	–0.90	1.30
Mortality in old adult males (*p*)	0.30 ± 0.16	–0.19	2.23
Mortality in old adult females (*p*)	0.22 ± 0.16	–0.56	0.80
Dispersal mortality in males (*p*)	0.45	–1.67	1.61
Dispersal mortality in females (*p*)	0.43	–1.42	1.33
Longevity (*v*)	9	1.73	–2.12
Catastrophe occurrence (*p*)	0.04	–0.84	1.05
Catastrophe severity (*p*)	0.42	–1.89	3.28

*p* = probability, *v* = absolute value. Reference values from [6] or calibrated to match an observed pattern in the population modelled here (indicated by *, see [4]). Measures of precision could not be assigned to values that represent proportions. Sensitivity = ratio of the relative change in *T*_m_ to the relative change in parameter value.

Our model thus appears to capture the essential characteristics of a real wild dog population and to be relatively robust to parameter uncertainty (see [4]). Collectively, this suggests that the model is structurally realistic [8] enough to place confidence in inferences about real wild dog populations based on modelling results.

### Quantifying establishment probability

To determine a critical initial number of packs (consisting of eight individuals each; [6]) to ensure establishment, we used the “Wissel plot” (formerly referred to as “ln(1 – *P*_0_) plot”; [1]). This plot is based on the equation $P_{0}\left(t\right)=\;1–c_{1}e^{–\omega_{1t}}$, which relates the probability of extinction by time *tP*_0_(*t*), to two constants, *c*_1_ and *ω*_1_. The former, *c*_1_, reflects the initial state of a population at time *t* = 0. If this state is in the range of states that can be observed in the established phase, *c*_1_ is equal to one; if a population initially is so small that it does not necessarily reach the established phase but might go extinct beforehand, *c*_1_ is smaller than one [1]. The other constant, *ω*_1_, is independent of the initial state of a population and describes the probability of extinction per short time interval in the established phase, which is constant. The inverse of this risk, *T*_m_ = 1/*ω*_1_, can be defined as the “intrinsic mean time to extinction” [1]. It describes the intrinsic persistence of a population in a given area and environment. For *c*_1_ = 1, *T*_m_ is equal to the arithmetic mean time to extinction that can be determined from repeated simulations starting from the same initial population [9].

**Figure 1:**
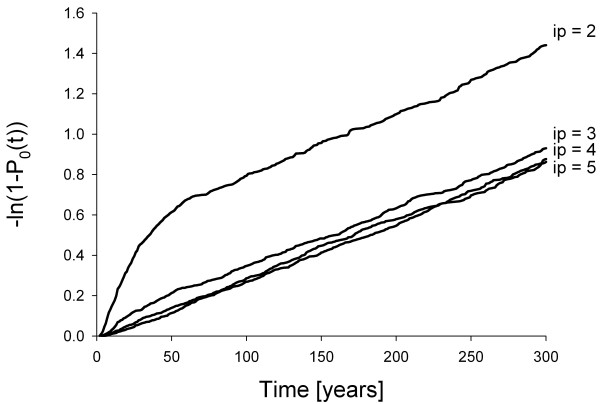
**“Wissel plots” (–ln(1 –*P***_**0**_**(*t*))*vs.*time*t*), each produced from 1000 simulations of a wild dog population model.** For parameters, see Table 1 (initial condition: number of packs (*ip*) = 2 to 5; probability for a disperser group to leave release area = 0.4). The slope of the plots, which is the inverse of the intrinsic mean time to extinction *T*_m_, is independent of *ip*, but *ip* affects the plots’ position and thus the intercept of the (extrapolated) linear parts of the plot with the *y*-axis, which is –ln(*c*_1_)

**Figure 2:**
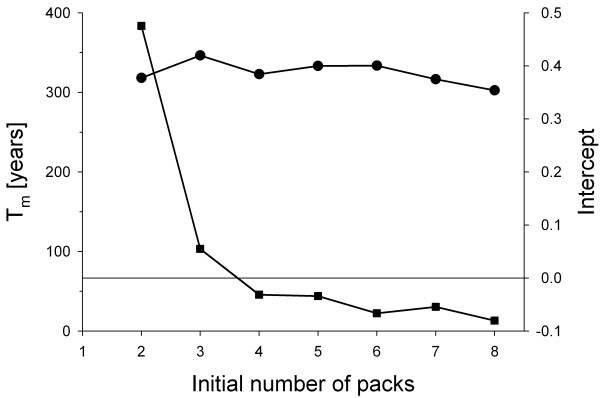
**Intrinsic mean time to extinction*T***_**m**_**of a reintroduced wild dog population (dots) and intercept of the “Wissel plots” with the*y*-axis (squares).** For parameters, see Table 1 (initial condition: number of packs = 2 to 8; probability for a disperser group to leave release area = 0.4). Only if the initial number of packs is four or larger, the *y*-intercept is zero or smaller and thus establishment of the population ensured. (*T*_m_ and the *y*-intercept were determined from simulated extinction times using a user-friendly software tool that is available from the authors upon request.)

The two constants *c*_1_ and *ω*_1_ can easily be determined by running, say, 1000 simulations, determining *P*_0_(*t*) by successively registering all extinction events by time *t*, and by using the “Wissel plot”, where –ln(1 – *P*_0_(*t*)) is plotted against time [1,9]. (A user-friendly software tool that takes extinction times from simulations as input, performs the “Wissel plot” and delivers *c*_1_ and *T*_m_ as output is available from the authors upon request.) The slope of the linear parts of all “Wissel plots” shown in Figure 1 is, as predicted from theory, the same and thus independent of the initial number of packs. Its inverse, the intrinsic mean time to extinction *T*_m_, is about 320 years in this case (Figure 2). (Note that releasing a single pack does not make sense because new packs can only emerge from disperser groups originating from different packs. Thus, in this case, all disperser groups leaving the pack are effectively lost and consequently *T*_m_ is only about 30 years.)

If the initial number of packs is larger than one but still too small, *c*_1_ is smaller than one and thus the intercept of the (extrapolated) linear parts of the “Wissel plot” with the *y*-axis, which is –ln(*c*_1_), is positive (Figure 1). Figure 2 shows that the *y*-intercept is positive if the initial number of packs is two or three. Thus, to ensure establishment, four packs should be released. As expected, this is more than the number of packs (two) necessary for a reintroduced population to maintain itself in the release area with frequent supplementation [4]. However, the extinction times presented in Figure 2 are very short. An intrinsic mean time to extinction *T*_m_ of 10,000 years corresponds to an extinction risk of 1% in 100 years [1], thus even the largest *T*_m_ obtained in our case results in an extinction risk exceeding 10%.

**Figure 3:**
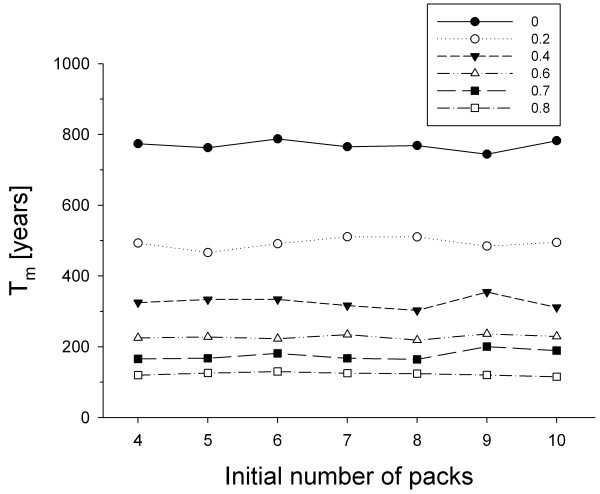
**Intrinsic mean time to extinction*T***_**m**_**of an established wild dog population.** For parameters, see Table 1 (initial condition: number of packs = 4 to 10; probability for a disperser group to leave release area = 0.0 to 0.8). A realistic emigration rate of 40% more than halves the population’s persistence

In wild dogs, new packs typically form when two unrelated opposite sex disperser groups meet and bond [10]. Theoretical models predict that this process could be limited by problems in finding suitable mates when population size is small [11], and we indeed found such a mate-finding Allee effect at low pack numbers in the wild dog population modelled here [6]. In this population, the proportion of disperser groups that failed to form a new pack was 41% (predicted) and 43% (observed), respectively [4]. Assuming that these disperser groups subsequently leave the release area in search of mates elsewhere [6], the population has to be able to withstand an emigration rate of about 40%. Such a high probability for a disperser group to leave the release area more than halves the population’s viability (Figure 3), suggesting a strong influence of emigration on the persistence of an established population in this case (cf. [12]).

The asymptotic nature of our results for establishment (Figure 2) is suggestive of a critical threshold size typical for an Allee effect (cf. [3,13]). From Figures 1 to 3 it becomes clear that it does not make sense to release more than four packs, because this neither increases population establishment nor promotes persistence. Strikingly, we previously established empirically that a critical minimum number of four packs, which simultaneously produce enough unrelated dispersers, are necessary for successful pack formation events to occur [6], and thus to maintain population viability. With a given number of individuals available for reintroduction, consecutive releases of several smaller packs (Figure 2 in [4]) result in higher population viability than a single release of a few larger packs (Figure 3), as this may buffer a newly founded population from environmental stochasticity, but such frequent supplementations may not be feasible.

Sensitivity analysis (Table 1) showed that reproduction (both the probability of producing a litter and litter size), and thus the production of future dispersers, most strongly affect population viability (cf. [14,15]). Focusing conservation management on enhancing reproduction thus seems particularly advisable in this case, and behavioural traits were indeed found to most strongly affect the survival of reintroduced wild dogs [16]. Variation in release pack size, on the other hand, had a small impact on population viability (Table 1), as we previously established empirically [6].

## Conclusions

Release strategies are often based on intuition and trial-and-error rather than a critical appraisal of the available evidence [17]. Reintroduction biology is a typical field where initial conditions are important: a population may not realize its intrinsic ability to persist because it is too small, and consequently goes extinct before establishment. The dichotomy between establishment and persistence is useful because newly founded populations can fail to reach the established phase in conditions that would enable persistence once the population is established [2].

Therefore, reintroduction biology and related disciplines (e.g. invasion and restoration ecology as well as the emerging field of assisted colonization) would likely benefit from using structurally realistic models as well as adopting the plot and concepts proposed by [1] to assess establishment separately from persistence (i.e. to differentiate between initial and intrinsic aspects). The method we present for doing this is generic and inferences thus are transferable to other systems. The plot of –ln(*c*_1_) in Figure 2 (intercept with *y*-axis) represents a novel application of the “Wissel plot” [1] to quantify the establishment probability of newly founded populations across the field of conservation biology. It should be emphasized, though, that the reliability of recommendations for real (re)introductions, which are based on the approach presented here, depends on the reliability of the underlying population model, which needs to be well documented, tested and validated.

## Competing interests

The authors declare that they have no competing interests.

## Authors’ contributions

All authors contributed to the design of the study, acquisition and interpretation of data, and writing of the manuscript. All authors read and approved the final manuscript.
